# Evaluation of a modified short all oral treatment regimen for rifampicin-multidrug resistant tuberculosis in Dominican Republic

**DOI:** 10.1186/s12879-024-10417-w

**Published:** 2025-02-09

**Authors:** María Rodríguez, Yamile Celis Bustos, Melanea Encarnación, Elisabet Muñoz, Sandra De los Santos, Ingrid Sánchez, Lissette Portorreal, Seydou Benjamín Sombie, Fatimata Bintou Sall, Corinne Simone Merle, Freddy Perez

**Affiliations:** 1 National Tuberculosis Program, Ministry of Health, Santo Domingo, Dominican Republic; 2 Department of Communicable Diseases Prevention, Control, and Elimination, Pan American Health Organization, Washington, DC USA; 3 DR-TB Regional Units, National Health Service, Santo Domingo, Dominican Republic; 4 Clinical Management Department, National Health Service, Santo Domingo, Dominican Republic; 5 Special Programme for Research and Training in Tropical Diseases (TDR), World Health Organization, Geneva, Switzerland; 6 Federal University of Health Sciences of Porto Alegre (UFCSPA), Porto Alegre, RS Brazil; 7 National TB Program, Ministry of Public Health of the Dominican Republic, Av. Dr. Héctor Homero Hernández, Esq. Av. Tiradentes, Ens. La Fe, 10514 Santo Domingo, Dominican Republic

**Keywords:** Tuberculosis, Multidrug-resistant, Bedaquiline, Linezolid, Short all-oral treatment, Treatment outcome, Safety, Health-Related Quality of Life, Dominican Republic

## Abstract

**Background:**

This study aims to evaluate the effectiveness, safety, and impact on health-related quality of life (HQoL) of a fully oral shortened regimen for Rifampicin-Resistant/Multidrug-Resistant Tuberculosis (RR/MDR-TB) over 9 to 12 months under programmatic conditions.

**Methods:**

A prospective cohort study was conducted on an all-oral modified Shortened Treatment Regimen (mSTR) comprising linezolid (Lzd), bedaquiline (Bdq), levofloxacin (Lfx), clofazimine (Cfz), and cycloserine (Cs). Patients with RR/MDR-TB were enrolled between January and December 2022 across seven drug-resistant TB units in the Dominican Republic.

**Results:**

A total of 113 patients were enrolled, with 87% achieving culture conversion at two months. Treatment outcomes revealed that 79% of patients were successfully treated and didn’t relapse six months after the end of the treatment, 14% were lost to follow-up during the treatment, 6% deceased, and one experienced treatment failure due to Adverse Drug Reactions (ADRs). Adverse events of Special interest (AESI) were common, with 82% of patients experiencing at least one AE with high proportion of QT interval prolongation, elevated transaminases, and anemia. A total of 12% of the patients experiencing Serious Adverse Events (SAEs). Improvement in HQoL dimensions was noted throughout treatment, with the EQ-VAS score increasing by an average of 15.5 by treatment end.

**Conclusion:**

The high treatment success rate of the 5-drug mSTR facilitated the adaptation and integration of a shortened treatment regimen lasting 9 to 12 months in routine care in Dominican Republic. SAEs were -rare. Although AESI were frequent, they were manageable in most cases. Continuous monitoring, particularly with regard to the use of Lzd and Bdq, is crucial to effectively mitigating risks. Since September 2023, this short all oral treatment regimen is the recommended approach for patients with RR/MDR-TB in the Dominican Republic.

**Supplementary Information:**

The online version contains supplementary material available at 10.1186/s12879-024-10417-w.

## Introduction

Tuberculosis (TB), one of the most prevalent infectious diseases globally, continues to exert a significant burden on mortality and morbidity. In 2022 TB ranked as the second- largest infectious killer worldwide, surpassed only by coronavirus disease (COVID-19) [1]. It was reported as the primary cause of death among people with the human immunodeficiency virus (HIV) and a significant contributor to deaths related to antimicrobial resistance. The COVID-19 pandemic and the measures adopted to control it significantly disrupted the detection, diagnosis, and treatment of TB and TB/HIV co-infection cases, while also causing substantial economic and social impact on the Dominican population [2]. In 2022, the global estimate for cases of multidrug resistant or rifampicin-resistant TB (RR/MDR-TB) was 410,000. In the Region of the Americas, approximately 12,000 cases of RR/MDR-TB were estimated, constituting an estimated proportion of 3.2% (2.1–4.4) of new cases, with 5,444 cases reported [3].

The Dominican Republic has consistently ranked among the top five countries with the highest MDR-TB rate in the Region of the Americas since the publication of the first national surveillance report in 1998 [4, 5]. With a drug susceptibility testing (DST) coverage of 89% and an estimated proportion of 6.8% (6-7.7) among new cases, the Dominican Republic reported 185 cases RR/MDR-TB out of 4,306 notified in 2022 [3].

Between 2006 and 2008, cohorts on standardized regimens comprised kanamycin, ofloxacin, ethionamide, cycloserine and pyrazinamide achieved a success rate of 74% at national level [6]. These regimens covered periods of 18–24 months, with injectable drugs administered for six months. In 2010, ofloxacin was replaced by levofloxacin and kanamycin by capreomycin [6] From 2014, the decentralization process and an outpatient model of care for RR/MDR-TB was introduced, maintaining regimens with injectables. However, there was a decline in treatment success from 74 to 60%, mainly attributed to high rates of patients lost to follow-up (28–35%) [7]. Following the World Health Organization (WHO) recommendation of an all-oral treatment regimen [8, 9], the success rate increased from 60 to 75% in 2020. This was due to a decrease of the lost to follow-up rate to 18% by 2020. Nevertheless, additional measures needed to be taken to further decrease the percentage of patients lost to follow-up and improve overall success rate for all patients [3, 10]. Shortening the length of the treatment was felt as a key strategy to consider.

Since 2020, the national TB control program (NTCP) of the Dominican Republic, in collaboration with the Special Programme for Research and Training in Tropical Diseases (TDR) of the World Health Organization (WHO) and the Pan American Health Organization (PAHO), has been conducting operational research on the effectiveness, safety, and impact on health-related quality of life (HQoL) of RR/MDR-TB patients treated with the modified Short all oral Treatment Regimen (mSTR) for drug-resistant TB patients [11].The introduction of a fully oral modified short treatment regimen holds significant potential to reduce the duration of treatment, enhance treatment compliance, and ultimately increase the success rate of treatment [3, 12].

The five-drug WHO-recommended STR has been in implementation since February 2022. Our objective was to evaluate the effectiveness, safety, and impact on HQoL of this 9 to 12 month fully oral shortened RR/MDR-TB under programmatic conditions. In this paper, we present end-of-treatment and follow up outcomes at three and six months after the end of the treatment, which could inform RR/MDR-TB treatment policy for Dominican Republic.

## Methods

### Study design

A prospective cohort study was conducted that enrolled patients with RR/MDR-TB between January and December 2022 in seven drug-resistant TB units in the Dominican Republic. The research protocol and data collection tools were developed using the Short all-Oral Regimens for Rifampicin-resistant Tuberculosis (ShoRRT) research package proposed by TDR [11], and adapted to the context of the country.

### Study population

The study population consisted of TB patients with evidence of resistance to at least rifampicin (R) as detected by the WHO-recommended rapid molecular diagnostic test for TB (mWRD), Xpert^®^ MTB/RIF Ultra, or conventional (culture-based) drug susceptibility testing (DST). For minors (18 years of age and younger), inclusion criteria involved confirm TB cases without DST, but with close contact with a confirmed RR/MDR-TB case. The total number of expected patients was based on those meeting the specified criteria in the country between January to December 2022. Patients from the different sites were included in the cohort as per convenience. Isoniazid (H) and Fluoroquinolone (FQ) susceptibility were assessed by Line Probe Assay (LPA) and/or phenotypic DST.

The main exclusion criteria included FQ resistance, inability to take oral medications, allergies, or the use of medications contraindicated with any of the drugs in the mSTR regimen, and a Fredericia-corrected QT interval (QTcF) of ≥ 500 ms at baseline that does not correct with medical treatment. Additionally, patients less than 2 years of age were excluded.

Patients who met the inclusion criteria and gave their consent were enrolled in the study as they presented at the health center for treatment during the study period.

### Study procedures

#### Short treatment regimen under investigation

The study regimen consisted of five drugs: linezolid (Lzd), bedaquiline (Bdq), levofloxacin (Lfx), clofazimine (Cfz), and cycloserine (Cs), at doses calculated by weight according to the recommendation of WHO [12] (supplementary table S1). The initial phase compromised four-months with Lzd-Bdq-Lfx-Cfz-Cs, followed by a two-month continuation phase with Bdq-Lfx-Cfz-Cs, and a three-month maintenance phase with Lfx-Cfz-Cs. The total treatment duration was 9 months with a possible extension to 12 months if the 4 -month sputum culture was positive. Patient follow-up continued for 12 months (3, 6 and 12 months) after the end of the treatment.

### Evaluation criteria

#### Effectiveness

the proportion of RR/MDR-TB patients with a favorable treatment outcome defined as cured or treatment completed without recurrence at 3, 6 and 12 months after the end of the treatment.

#### Safety

the proportion of RR/MDR-TB patients who experience a serious adverse event up to six-months after the end of the treatment.

#### Health-related quality of life (HQoL)

HQoL was evaluated using the Euroquol EQ-5D-5 L questionnaire [13], a concise, generic measure of self-reported health status. The assessment focused on determining the proportion of patients who experienced an improvement in their quality of life during the treatment period.

### Investigation plan

To evaluate the effectiveness, safety, and impact on the HQoL of the shorter oral treatment regimen implemented in this study, the following parameters were monitored monthly during treatment and six months after the end of the treatment: baseline patient characteristics, clinical evaluation, bacteriology (DST, smear, and culture), laboratory tests (particularly Hemoglobin level), and additional parameters detailed in supplementary Table S2.

### Data collection and analysis

Data was collected using standardized forms built on the REDCap electronic data capture tool hosted at WHO Geneva [14]. Periodic data cleaning for identifying missing data and inconsistences was performed centrally by the coordinating center of the Dominican Republic national TB programme. Data were collected for the following variables: age in years, gender (male, female), TB treatment history, TB type (pulmonary or extra-pulmonary); TB category (new or previously treated); sputum smear result (smear-positive, smear-negative, smear not done); Mycobacterium tuberculosis culture result (positive, negative, not done); drug susceptibility testing (DST) results if available; treatment start date; sputum culture conversion (Yes, No); date of sputum culture conversion; para-clinical parameters (chest X-ray, electrocardiogram, blood test); final treatment outcome (cured, treatment completed, treatment failed, died, lost to follow up and not evaluated) as defined by WHO [15]; TB recurrence; adverse events and HQoL data.

The occurrence of SAEs and Adverse Events of Special Interest (AESI) and adverse drugs Reaction (ADR) were monitored and reported monthly in accordance with the recommendations of the WHO and the research protocol ShoRRT of TDR [15, 16]. In our study we define ADR as a response to a TB medicine that is noxious and unintended, and which occurs at doses normally used in humans [17].These events included blood-related issues, cardiac concerns (such as QTcF prolongation), hepatic complications (Alanine aminotransferase enzyme increase), nervous system disorders (including optic neuritis, peripheral neuropathy) and mental disorders. The severity of adverse events was graded according to the Division of AIDS table for grading the severity of adult and pediatric patients, as outlined by the U.S. National Institutes of Health [18] and for QTc prolongation, the National Cancer Institute Common Terminology of Clinical Adverse Events v5 was utilized [19].

For HQoL assessment, data were collected at enrolment, four months into treatment, at the end of treatment and 12 months after the end of treatment, using the EQ-5D-5 L questionnaire [20]. This questionnaire comprises two parts: the EQ-5D-5 L, which includes a descriptive system of five dimensions (mobility, self-care, usual activities, pain/discomfort, and anxiety/depression) in five levels. Participants indicated for each dimension whether they have no, mild, moderate, severe, or extreme problems. For each dimension, the proportion of patients with no, mild/moderate and severe/extreme problems was determined at each time point. The second part is the EQ-VAS which captures the respondent’s overall assessment of their health on a scale from 0 (worst health) to 100 (best health).

Categorical variables were described using frequencies and percentage, while continuous variables were summarized using means with standard deviation or medians with 1st and 3rd quartiles. For HQoL data, data analysis, changes in health states for each dimension were determined at four months into treatment and at the end of treatment, alongside mean EQ-VAS scores at baseline and the average difference EQ-VAS score from baseline. Data analyses were performed using STATA software (version 17).

### Ethics approval

The study received ethical approval from the WHO Research Ethics Review Board (Generic Protocol ID: ERC0003305, 08/06/2020) and the Ethics Committee of the Dr. Hugo Mendoza National Center for Research in Maternal and Child Health (CENISMI, 10/07/21). Informed consent was obtained from each participant or respondent. For participants under the age of 16, consent to participate was obtained from their parents or legal guardians. All methods adhered to relevant guidelines and regulations.

## Results

**Fig. 1:**
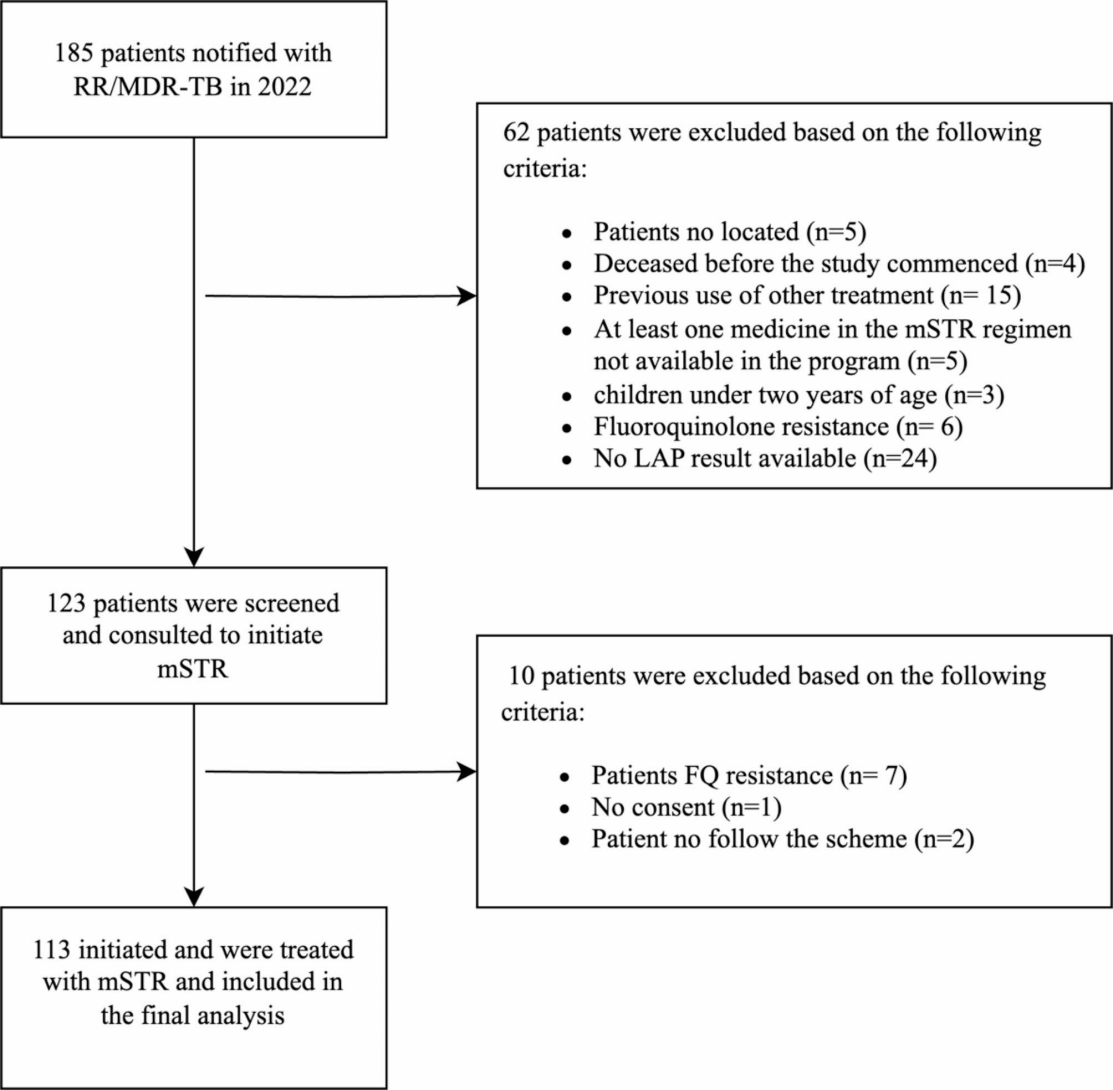
Flow diagram of eligible study participants from RR/MDR-TB notified patients in 2022

Between January to December 2022, a total of 185 patients with RR/MDR-TB were diagnosed using Xpert MTB/RIF Ultra in seven drug resistant TB units. Sixty-two patients were excluded due to being unlocatable or deceased before treatment initiation, prior use of drugs from the regimen or not available at the program, age under two years, absence of LPA results, known FQ-resistance, or the presence of cardiovascular complications with QTcF > 500ms. Of the remaining 123 patients, screening and consultation were conducted for potential inclusion in the modified short treatment regimen (mSTR). Seven were further excluded due to FQ-resistance identified with second-line drug susceptibility testing (SL-DST), two never initiated the mSTR and one withdrew consent (Fig. 1).

### Baseline characteristics of patients

A total of 113 patients were included in the effectiveness analysis. among these patients, 80 were male (71%), with a median age of 40 years, and 74 patients (65%) were aged between 25 and 55 years. Table 1 presents the baseline characteristics of these patients.

**Table 1:** Demographic and clinical characteristics of patients with RR/MDR-TB included in the modified short treatment regimen cohort in Dominican Republic

Characteristics	Total (*N* = 113)	95% CI^1^
**Gender**	*n* (%)	
Male	80(71%)	[61–79]
Female	33(29%)	[21–39]
**Age in years**	**n (%)**	
mean (SD)	40(15)	[38–43]
15–24	16(14%)	[9–22]
25–55	74(65%)	[56–74]
> 55	23(21%)	[14–29]
**Type of patient** ^**2**^	**n (%)**	
New case	85(75%)	[66–83]
Relapse	16 (14%)	[9–22]
Treatment after LFTU^3^	6 (5%)	[2–12]
Treatment after failure	3(3%)	[1–8]
Other treatment	3(3%)	[1–8]
**Disease site**	**n (%)**	
Pulmonary	113(100%)	[96–100]
**Tuberculosis treatment history**	**n (%)**	
Previously treated with only first-line drugs^4^	28 (25%)	[17–34]
**Co-morbidities**	**n (%)**	
HIV positive	14 (12%)	[7–20]
Hepatitis C positive	1 (1%)	[0–7]
Diabetes positive	18 (16%)	[10–24]
Existing neuropathy	7(6%)	[3–13]
**Bacteriological characteristics**	**n (%)**	
Positive sputum culture	75 (66%)	[57–75]
Rifampicin resistant	112(98%)	[94–100]
Isoniazid resistant	67 (59%)	[50–68]
Fluoroquinolone sensitive^5^	72(64%)	[54–72]
Fluoroquinolone sensitivity Unknown	41 (36%)	[28–46]
**Chest X-Ray**	**n (%)**	
Cavity	71 (63%)	[55–74]
**Extend of the disease** ^**6**^	**n (%)**	
A (< 25%)	41(36%)	[28–46]
B (25–49%)	43 (38%)	[29–48]
C (≥ 50%)	25(22%)	[15–31]
**Laboratory and Clinical Parameters**	**median (Q1-Q3)** ^7^	
**Hematological evaluation**		
Hemoglobin (g/dL)	11.8 (10.4–13.2)	[11–12]
Platelet (10^9^/L)	396 (291–470)	[365–432]
White blood count (10^9^/L)	9.02(6.8–11.8)	[8.9–11]
**Liver and renal function**		
AST/SGOT (U/L)	24 (17–36)	[26–34]
Creatinine (mg/dL)	0.83 (0.7-1)	[0.81–0.89]
**Clinical evaluation**		
BMI in kg/m²	20.25 (17.95–23.2)	[20–23]
QTcF Intervals (ms)	396 (355–437)	[378–406]
**Health Quality of life (EQ-5D)**	**n (%)**	
Mobility		
Slight/moderate problems	14(16%)	[7–20]
Severe problems / Unable	2(2%)	[0–7]
Self-care		
Slight/moderate problems	4(5%)	[1.1–9.3]
Severe problems / Unable	1(1%)	[0–6]
Usual activities		
Slight/moderate problems	15(17%)	[8–21]
Severe problems / Unable	2(2%)	[0–7]
Pain/Discomfort		
Slight/moderate problems	14(16%)	[7–20]
Severe problems / Unable		
Anxiety/Depression		
Slight/moderate problems	1(1%)	[0–6]
Severe problems / Unable	15(17%)	[8–21]
**General health assessment (EQ-VAS score)**		
Mean (SD)	79 (14)	[76–83]

^1^ 95% Confidence Interval; For numerical variables, 95% CI of the mean is presented

^2^Definitions based on the recommendations of the World Health Organization (WHO) [9]

^3^LFTU: Lost to follow-up

^4^Two previously treated cases with missing information

^5^Information obtained from LPA and DST

^6^ A: less than 25% lung parenchyma affected; B: Between 25% and 49% of lung parenchyma affected; C: 50% or more of the lung parenchyma affected

^7^Q1 = 1st quartile; Q3 = 3rd quartile

All patients had confirmed pulmonary rifampicin resistant TB. Among them, 85 (75%) were new patients, while 28 (25%) had prior treatment with first-line drugs, including 16 (14%) classified as relapses. Fourteen patients (12%) were tested positive for HIV, 18 (16%) had diabetes and 7 (6%) had existing neuropathy. A positive sputum culture was observed in 75 out of 113 patients (66%), with DST results available for 72(64%), with 67 being resistant to isoniazid (59% MDR-TB). Seventy-two (64%) patients had DST results confirming fluoroquinolone susceptibility. Among all patients, 71 (63%) had TB cavities, and 25 (22%) had extended lesions (≥ 50%) on chest X-Ray (Table 1).

The hematological evaluation revealed a median hemoglobin level of 11.8 g/dL (10.4–13.2) g/dL. Twenty-seven (24%) patients had grade 1 or more anemia (less than 10.5 g/dl) at baseline. Liver function test was within the normal range (AST/SGOT 24(17–36) U/L), except for one patient. No initial alteration of renal function was observed (creatinine 0.83 (0.7-1) mg/dl). The mean value of the body mass index (BMI) was 20.25 (17.95–23.2) kg/m² and the mean value QTcF intervals was 396 (355–437) ms.

Regarding HQoL, patients reported problems with physical functioning including mobility (18%), self-care (6%) and usual activity (19%). Additionally, 17% of surveyed patients reported problems with pain/discomfort domain, while anxiety/ depression was reported by 19% of participants. The EQ-VAS score mean was 79 (± 14),

### Treatment outcome

**Fig. 2:**
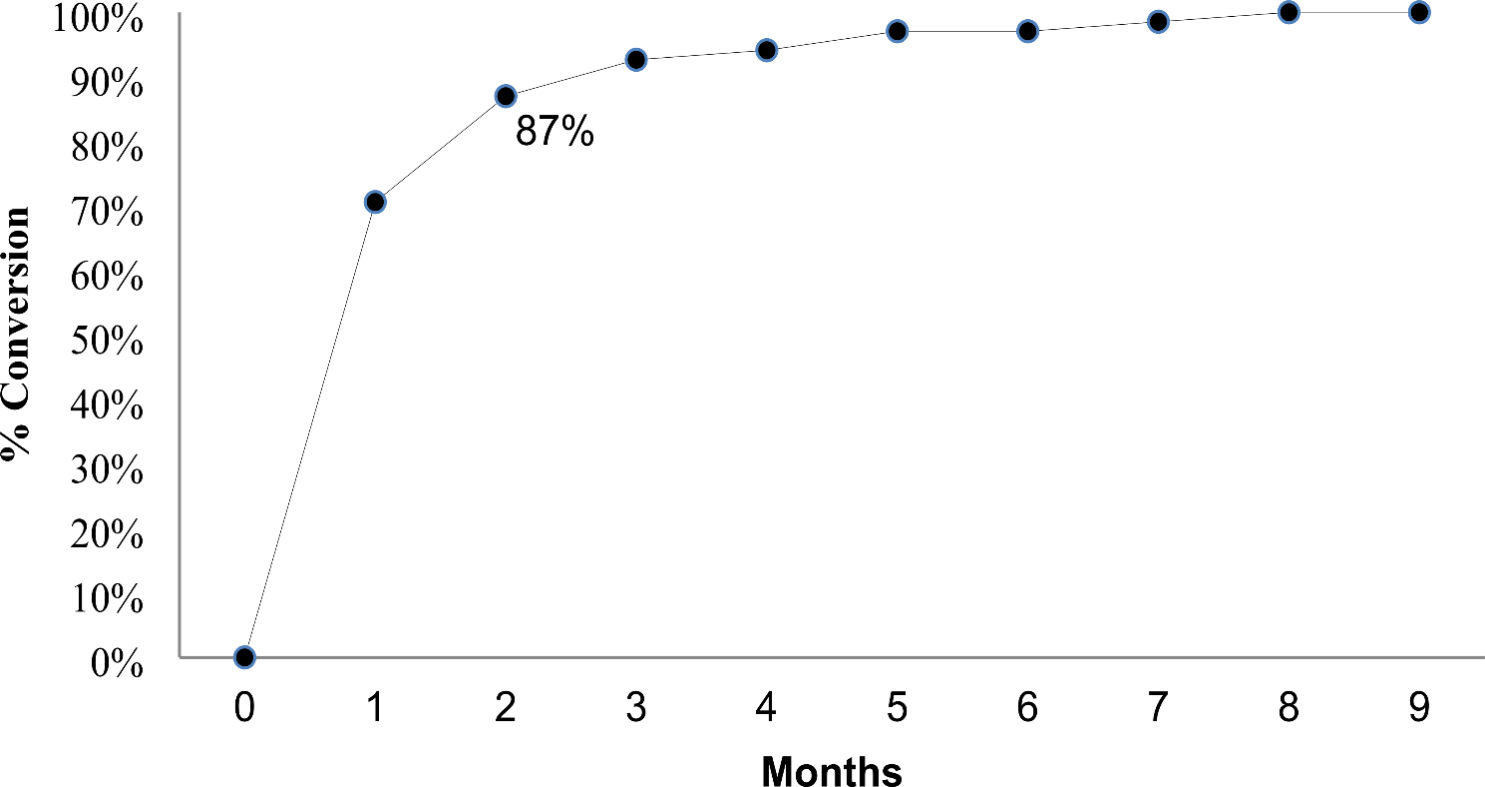
Time to Culture Conversion in patients undergoing mSTR for RR/MDR-TB

Among 113 patients, 89 (79%) had a favorable outcome, defined as being successfully treated without recurrence six months after the end of the treatment. Sixteen patients (14%) were lost to follow up, with three of them lost before completing three months of treatment, and two of these individuals tested negative in cultures. Seven patients (6%) died, and one experienced treatment failure, resulting from a permanent regimen change due to Adverse Drug Reactions (ADRs) (Table 2). Of the 41 patients who did not have fluoroquinolones’ DST results, 34 (83%) had a favorable outcome, three (7%) were lost to follow up and four (10%) died. Among the total patients, 75 had a positive culture at baseline, and 69 had a follow up smear result. Culture conversion in the second month was achieved for 60 patients (87%) (Fig. 2).

**Table 2:** Treatment outcomes after six months follow up for patients undergoing the modified short treatment regimen cohort in Dominican Republic

Treatment outcome^1^	Total (*n* = 113) *n* (%)	95% CI
***Favorable*** ^**2**^	89 (79%)	[70–86]
**Unfavorable**	24 (21%)	[14–30]
Died during the treatment	7 (6%)	[3–13]
Lost to follow up	16 (14%)	[9–22]
Treatment failed^3^	1 (1%)	[0–6]
Relapse at 6 months follow up	0 (0%)	[0–4]

^1^Definitions of TB treatment outcomes for Patients with RR/MDR-TB based on the recommendations of the World Health Organization (WHO) [9]

^**2**^Favorable include successfully treated without recurrence 6 months after the end of the treatment

^3^Permanent change of at least 2 drugs because of Adverse Drug Reactions (ADR)

A favorable outcome was obtained with a treatment duration of nine months for 82 (92%) patients, 11 months for 5 patients and 12 months for 2 patients. The reasons for extending treatment beyond nine months were a positive sputum smear at four months of treatment in 3 patients and difficulties in compliance in 4 patients.

### Safety aspects

Out of 113 patients, 93 (82%) experienced at least one AESI. Fourteen patients (12%) had at least one SAE which resulted in death for seven of them. A total of six SAEs were ADR related to TB drugs. None of the ADRs resulted in death. A total of 237 AESI were recorded, with 106 (45%) classified as grade 1, 102 (43%) as grade 2, and 27 (11%) as grade 3. Two (1%) of the AESI were Grade 4. The most frequent AESI included prolonged QTcF in 119 (50%) cases, elevated transaminases in 56 (23%), anemia in 41 (17%), and thrombopenia in 11 (5%). Peripheral neuropathy was found in 6 patients (3%) and none of the patients suffered from optic neuropathy. More details can be found in the Table 3.

**Table 3:** Serious adverse events, adverse events of interest and grading in patients undergoing the modified short treatment regimen cohort in Dominican Republic

Type of Adverse Events	Total of patient (*n* = 113)	
	*n*	% [95% CI]
**Serious Adverse Events**		
**Number of patients who had at least one serious adverse event**	14	12 [7–20]
**Type of SAE**	**Total of events (** *n* ** = 15)**	
- Death^1^	7	47 [22–73]
- Life-threatening experience	2	13 [2–41]
- Hospitalization/prolongation of hospitalization	2	13 [2–41]
- Persistently/significantly disabling events	0	0 [0–25]
- Congenital anomalies/birth defects	0	0 [0–25]
- Other medically important events	4	27 [9–55]
**SAE related to use of medications**	**Total of events (** *n* ** = 15)** ^**2**^	
- Related	6	40 [17–67]
- Not related	7	47 [22–73]
- Insufficient data to assess	2	13 [2–41]
**Adverse event of interest**		
**Had at least one adverse event of interest**	93	82 [74–89]
**Type of AE of interest and their grading** ^**3**^	**Total of event (** *n* ** = 237)**	
- **Anemia**	**41**	**17** [13–23]
Grade 1	24	59 [42–73]
Grade 2	9	22 [11–38]
Grade 3	7	17 [8–33]
Grade 4	1	2 [0–14]
- **Thrombopenia**	**11**	**5** [2–8]
Grade 1	7	64 [32–88]
Grade 2	3	27 [7–61]
Grade 3	1	9 [0–43]
- **Leukopenia**	**2**	**1** [0–3]
Grade 1	2	100 [20–100]
- **Elevated liver enzymes**	**56**	**23** [18–30]
Grade 1	47	84 [71–92]
Grade 2	8	14 [7–27]
Grade 4	1	2 [0–11]
- **Prolonged QtcF**	**119**	**50** [44–57]
Grade 1	22	19 [12–27]
Grade 2	78	65 [56–74]
Grade 3	19	16 [10–24]
- **Peripheral neuropathy**	**6**	**3** [1–6]
Grade 1	3	50 [18–81]
Grade 2	3	50 [18–81]
- **Anxiety**	**2**	**1** [0–3]
Grade 1	1	50 [9–90]
Grade 2	1	50 [9–90]
- **Optic neuritis**	**0**	**0** [0–2]
**Proportion of patients who experienced each AE of interest**	**Total of patient (** *n* ** = 113)**	
- Anemia	30	27 [19–36]
- Thrombopenia	8	7 [3–14]
- Leukopenia	1	1 [0–6]
- Elevated liver enzymes	41	36 [26–46]
- Prolonged QtcF^4^	77	68 [59–76]
- Peripheral neuropathy	6	5 [2–12]
- Anxiety	2	2 [0–7]
- Optic neuritis	0	0 [0–4]
**Number of episodes of AE of interest per patient**	**Total of patient (** *n* ** = 93)**	
- Mean (SD)	3 (2)	
- Median (IQR)	2 (1, 3)	
- Minimum	1	
- Maximum	8	

^1^ Deaths unrelated to medication use

^2^ One patient had two SAE

^3^The severity of adverse events was graded according to the Division of AIDS table for grading the severity of adult and pediatric patients, as outlined by the U.S. National Institutes of Health [14]^10^

^4^The analysis of QTc prolongation utilized the National Cancer Institute Common Terminology of Clinical Adverse Events (CTCAE) v5.013

### Health related quality of life (HQoL)

In all five dimensions, most patients who reported problem at baseline and were evaluated after four months of treatment felt better (73% for mobility, 50% for self-care, 69% for usual activities, 71% for pain/discomfort and 92% for anxiety/depression). At the end of treatment, almost all evaluated patients who had reported problem at baseline felt better (100% for the dimensions mobility and usual activities; 91% for the dimensions pain/discomfort and anxiety/depression). In the dimension of self-care, only two patients with problem at baseline were evaluated with one showing improvement and one showing worsening (Table 4).

**Table 4:** Evaluation of health quality of life during the treatment phase among patients who reported at least one problem during the two-time periods: four months and end of treatment

HQoL evaluation*	Baseline − 4 month^*^ n (%) [95% CI]	Baseline - End of treatment^*^ n (%) [95% CI]
**EQ-5D-5 L**		
**Mobility**	*n* = 11	*n* = 8
Any problem - No change	1 (9.1%) [0–43]	0 (0%) [0–40]
Better	8 (72.7%) [39–93]	8 (100%) [60–100]
Worse	2 (18.2%) [3–52]	0 (0%) [0–40]
**Self-care**	*n* = 4	*n* = 2
Any problem - No change	0 (0%) [0–60]	0 (0%) [0–80]
Better	2 (50.0%) [15–85]	1 (50.0%) [9–90]
Worse	2 (50.0%) [15–85]	1 (50.0%) [9–90]
**Usual Activities**	*n* = 13	*n* = 10
Any problem - No change	1 (7.7%) [0–38]	0 (0%) [0–34]
Better	9 (69.2%) [39–90]	10 (100%) [66–100]
Worse	3 (23.1%) [6–54]	0 (0%) [0–34]
**Pain / Discomfort**	*n* = 14	*n* = 11
Any problem - No change	1 (7.1%) [0–36]	0 (0%) [0–32]
Better	10 (71.4%) [42–90]	10 (90.9%) [57–99]
Worse	3 (21.4%) [6–51]	1 (9.1%) [0–43]
**Anxiety/Depression**	*n* = 13	*n* = 11
Any problem - No change	0 (0%) [0–28]	0 (0%) [0–32]
Better	12 (92.3%) [62–100]	10 (90.9%) [57–99]
Worse	1 (7.7%) [0–38]	1 (9.1%) [0–43]
**EQ-VAS score**	*n* = 59	*n* = 58
Mean difference (SD) [95% CI]	3.3 (9.0) [0.95–5.64]	15.5 (14.0) [11.8–19.2]

*HQoL= Health Quality of Life; ** 59 patients had an HQoL evaluation both at baseline and after 4 months of treatment and 58 patients had an HQoL evaluation both at baseline and at the end of the treatment. For each domain of mobility, only patients who reported at least one problem during the two−time point that are compared are presented, the remaining patients did not report any problem neither at baseline, nor after four months or at the end of the treatment

Regarding the EQ-VAS score, the mean was, 84 (± 10.9) at four months of treatment and 96 (± 4) at the end of treatment. The mean difference in EQ-VAS score compared to baseline measurement was 3.3(± 9) at four months and 15.5 (± 14) at the end of the treatment (Table 4).

## Discussion

This study represents the first investigation conducted in Dominican Republic (DR) to assess the effectiveness, safety, and impact on the health-related quality of life (HQoL) among RR/MDR-TB patients treated with 9–12 months short all oral treatment.

The 5-drug all-oral modified short treatment (mSTR) implemented in this study mirrored the long regimen endorsed by the WHO [15]. It demonstrated a high early conversion rate (87% at 2 months) and a notable treatment success rate (79%) among RR/MDR-TB patients. This treatment success rate is comparable to the one achieved with the longer treatment regimen with the seven-drug STR containing injectable agents for the 2020 MDR-TB cohort (75%) [5]. Notable, none of the cured patients experienced relapse six months after the end of treatment, indicating sustained treatment cure [10].

Our results align with success rates (ranging from 75 to 90%) reported in other countries for RR/MDR-TB patients treated with all oral mSTR [21, 22]. Importantly, the treatment success achieved with this simplified mSTR, utilizing Bdq, Lfx and Lzd as core drugs, and Cfz and Cs as companion drugs, appears to provide more favorable outcomes than those found in Central Asia. For instance, In Uzbekistan, a cohort of 95 patients achieved only a 66% success rate using seven-drugs regiment with high dose of H and Mfx as core drug, and without Bdq [23].

Despite the lack of information on FQ susceptibility in 46 (41%) of the 113 patients, a conversion rate of 87% was achieved at two months. The high conversion and treatment success rate of the mSTR evaluated in this study may be attributed to the potent bactericidal and sterilizing activities of the two main drugs, Bdq and Lfx. Indeed, the use of Lzd has been associated with improved treatment outcomes, as reported by Nguyen in 2023 [21, 24].

Despite a decrease in the percentage of patient’s loss to follow-up in the Dominican Republic compared to previous years, dropping from 18% in 2020 to 14% in this study, there remains a need to strengthen a patient-centered care approach to further improve treatment compliance [25]. Analysis of the baseline and control culture results of patients lost to follow-up indicated a low risk of disease transmission to the community and/or development of bedaquiline resistance [26]. Only one patient’s culture status could not be determined. No cases of bacteriological failure were recorded. Treatment modification was necessary for only one patient due to adverse events related to medication use. Seven patients died during treatment from causes unrelated to medication use.

Although adverse events (AEs) were common during RR/MDR-TB treatment, most of them were grade 1–2, consistent with findings from other studies [27, 28]. The most frequent AESI was prolonged QTc interval, reported in 68% of cases, which has been documented in other studies and associated with the use of Bdq and Cfz [28–30].

A total of fourteen patients (12%) experienced SAEs, with drug-related SAEs observed in six patients accounting for 40% of all SAE. Only one patient required a modification in the RR/MDR-TB regimen due to elevated transaminase levels associated with the use of Bdq and Lzd [12, 28]. Grade 3 and 4 anemia- was observed only in eight patients and was associated to the use of Linezolid (Lzd). Anemia was identified in a lower number of patients in this study compared to other studies reported where higher doses of Lzd were used [31]. This success may be attributed to the doses of Lzd used in this study (600 mg), which have been demonstrated to mitigate the toxicity associated with this drug [32]. From a national TB programme perspective, this AE seems to be manageable if carefully monitored.

In this study, patients started the treatment with favorable overall conditions (Table 1), including disease extent, clinical parameters and HQoL evaluations. This may be attributed to early diagnosis facilitated by the utilization of more sensitive rapid molecular tests.

The health-related quality of life EQ-VAS score in patient with RR/MDR-TB increased by an average of 16 points at the end of the treatment compared to baseline, indicating a significant enhancement in patients’ quality of life throughout the treatment period.

These results underscore the high effectiveness of the mSRT used in this study for treating patients with RR/MDR-TB in Dominican Republic. This regimen’s success enabled the shortening of the standardized treatment regimen, which originally had a longer duration, and facilitated an assessment of its impact on patient quality of life. The study demonstrated that this regimen does not impose a negative impact and, in fact, enhances treatment compliance.

One of the primary limitations of this research was the limited availability of fluoroquinolone molecular susceptibility testing. This constraint posed challenges in expanding the patient sample, emphasizing the importance of ensuring comprehensive coverage of this diagnostic test for all individuals with RR/MDR TB. Additionally, data concerning adverse events was collected from each patient through a specific tool for this purpose, nevertheless, only AESI were analysis for this study.

Safety profile appears manageable in most patients, with few serious adverse events observed, and very few attributed to medication use. Active Drug Safety Monitoring (aDSM) has been implemented in the Dominican Republic since begin of programmatic MDR-TB management; however, this study emphasizes the necessity to systematize and enhance aDSM in the country.

## Conclusion

This prospective study facilitated the evaluation and integration of a shortened treatment regimen lasting 9 to 12 months, utilizing the same drugs as the prolonged treatment regimen proposed by the WHO, tailored to the conditions in the Dominican Republic. This is shown by a high percentage of patients who were successfully treated. Although AESI were frequent, they were manageable, and SAEs were rare. Additionally, improvement in HQoL dimensions throughout the treatment was documented.

This allowed for the adjustment of national guidelines, providing an alternative approach for managing patients with RR/MDR-TB in the country. Furthermore, the implementation of this regimen resulted in improved patient compliance due to its shorter duration. Since September 2023, this short all oral treatment regimen is now the one recommended for RR/MDR-TB patients in Dominican Republic.

## Electronic supplementary material

Below is the link to the electronic supplementary material.

Supplementary Material 1

## Data Availability

Data is available within the manuscript.

## Abbreviations

COVID-19: Coronavirus disease
HIV: immunodeficiency virus
TB: Tuberculosis
MDR-TB: Multidrug-Resistant Tuberculosis
RR/MDR-TB: Rifampicin-Resistant/Multidrug-Resistant Tuberculosis
mSTR: Modified Shortened Treatment Regimen
ShoRRT: Short all-Oral Regimens for Rifampicin-resistant Tuberculosis
Lzd: Linezolid
Bdq: Bedaquiline
Lfx: Levofloxacin
Cfz: Clofazimine
Cs: Cycloserine
R: Rifampicin
H: Isoniazid
FQ: Fluoroquinolone
DST: Drug Susceptibility Testing
mWRD: molecular WHO-recommended Rapid Diagnostic test for TB
LPA: Line Probe Assay
ADRs: Adverse Drug Reactions
AEs: Adverse events
AESI: Adverse events of Special Interest
SAEs: Serious Adverse Events
QTcF: Fredericia-corrected QT interval
HQoL: health-related quality of life
NTCP: National Tuberculosis Control Programme
TDR: Special Programme for Research and Training in Tropical Diseases
WHO: World Health Organization
PAHO: Pan American Health Organization
