# Supplementary material for: Evaluation of a modified short all oral treatment regimen for rifampicin-multidrug resistant tuberculosis in Dominican Republic
Source: BMC Infect Dis. 2025 Feb 9;25:196. doi: 10.1186/s12879-024-10417-w (PMC11808990; doi:10.1186/s12879-024-10417-w)
Supplement: Supplementary file 1 — Supplementary Material 1 [file 12879_2024_10417_MOESM1_ESM.docx]

**Supplementary Data**

**Table S1.** Weight-based dosing of medicines used in Modified Short Treatment Regimen (mSTR) in this study.

| Drug | Formulation | 16–<24 kg | 24–<30 kg | 30–<36 kg | 36–<46 kg | 46–<56 kg | 56–<70 kg |  |  |  |
| --- | --- | --- | --- | --- | --- | --- | --- | --- | --- | --- |
| Levofloxacin (Lfx) | 500 mg tab |  | 1 | 1.5 | | 2 | |  |  |  |
| Bedaquiline (Bdq) | 100 mg tab | 2 od for 2 weeks; then 1 od M/W/F | | 4 od for 2 weeks; then 2 od M/W/F | | | | | |  |
| Linezolid (Lzd) | 600 mg tab | 0.5 | 0.5 | 0.5 | 0.75 | 1 | |  |  |  |
| Clofazimine (Cfz) | 100 mg tab | 1 M/W/F | 1 | | | | | | | |
| Cycloserine (Cs) | 250 mg tab | 2 | | | | 3 | | |  |  |

Tab: tablet, od: once daily, M/W/F: Monday, Wednesday, and Friday. Table taken and adapted from Module 4 of the WHO operational handbook on tuberculosis[9].

**Table S2**. Parameters monitored for Patients with RR/MDR-TB Undergoing the Modified Short Treatment Regimen (mSTR).

| **Observation** | **Baseline assessment and screening** | **Treatment Phase** | | | | | | | | | **Follow-up** | | | |  |
| --- | --- | --- | --- | --- | --- | --- | --- | --- | --- | --- | --- | --- | --- | --- | --- |
|  |  | **MT1** | **MT2** | **MT3** | **MT4** | **MT5** | **MT6** | **MT7** | **MT8** | **MT9/12** | | **MF3** | **MF6** | **MF12** | |
| **Clinical evaluation** | |  |  |  |  |  |  |  |  |  | |  |  |  | |
| Demographics, Medical History | X |  |  |  |  |  |  |  |  |  | |  |  |  | |
| Clinical Examination | X | X | X | X | X | X | X | X | X | X | | X | X | X | |
| Written informed consent | X |  |  |  |  |  |  |  |  |  | |  |  |  | |
| Treatment adherence |  | X | X | X | X | X | X | X | X | X | |  |  |  | |
| Concomitant treatment |  | X | X | X | X | X | X | X | X | X | | X | X |  | |
| Adverse events |  | X | X | X | X | X | X | X | X | X | | X | X |  | |
| **Bacteriology** |  |  |  |  |  |  |  |  |  |  | |  |  |  | |
| Sputum smear | X | X | X | X | X | X | X | X | X | X | | X | X | X | |
| Sputum culture | X | X | X | X | X | X | X | X | X | X | | X | X | X | |
| DST (FQ/Injectables) | X |  |  |  |  |  |  |  |  |  | |  |  |  | |
| **Laboratory tests** |  |  |  |  |  |  |  |  |  |  | |  |  |  | |
| Complete blood count | X | X | X | X | X | X | X | X | X | X | | X |  |  | |
| Serum creatinine^1^ | X |  |  |  |  |  |  |  |  |  | |  |  |  | |
| Serum potassium^1^ | X |  |  |  |  |  |  |  |  |  | |  |  |  | |
| Serum liver enzymes | X | X | X | X | X | X | X | X | X | X | |  |  |  | |
| Pregnancy test | X |  |  |  |  |  |  |  |  |  | |  |  |  | |
| HIV and hepatitis test | X |  |  |  |  |  |  |  |  |  | |  |  |  | |
| ECG | X | X | X | X | X | X | X | X | X | X | |  |  |  | |
| Visual acuity & BPNS^2^ | X | X | X | X | X | X | X | X | X | X | |  |  |  | |
| Health-related Quality of Life | X |  |  |  | X |  |  |  |  | X | |  |  | X | |

^1^At baseline and if clinically indicated or ECG abnormalities.

^2^BPNS - Baseline Peripheral Neuropathy Symptoms
